# Exome arrays capture polygenic rare variant contributions to schizophrenia

**DOI:** 10.1093/hmg/ddv620

**Published:** 2016-01-05

**Authors:** A. L. Richards, G. Leonenko, J. T. Walters, D. H. Kavanagh, E. G. Rees, A. Evans, K. D. Chambert, J. L. Moran, J. Goldstein, B. M. Neale, S. A. McCarroll, A. J. Pocklington, P. A. Holmans, M. J. Owen, M. C. O'Donovan

**Affiliations:** 1 MRC Centre for Neuropsychiatric Genetics and Genomics, Institute of Psychological Medicine and Clinical Neurosciences, Cardiff University School of Medicine, Hadyn Ellis Building, Cardiff CF24 4HQ, UK,; 2 Icahn School of Medicine at Mount Sinai, 1468 Madison Ave, New York, NY 10029, USA,; 3 Stanley Center for Psychiatric Research, Broad Institute of MIT and Harvard, Cambridge, MA, USA and; 4 Analytical and Translational Genetics Unit, Massachusetts General Hospital, Boston, MA, USA

## Abstract

Schizophrenia is a highly heritable disorder. Genome-wide association studies based largely on common alleles have identified over 100 schizophrenia risk loci, but it is also evident from studies of copy number variants (CNVs) and from exome-sequencing studies that rare alleles are also involved. Full characterization of the contribution of rare alleles to the disorder awaits the deployment of sequencing technology in very large sample sizes, meanwhile, as an interim measure, exome arrays allow rare non-synonymous variants to be sampled at a fraction of the cost. In an analysis of exome array data from 13 688 individuals (5585 cases and 8103 controls) from the UK, we found that rare (minor allele frequency < 0.1%) variant association signal was enriched among genes that map to autosomal loci that are genome-wide significant (GWS) in common variant studies of schizophrenia genome-wide association study (*P*_GWAS_ = 0.01) as well as gene sets known to be enriched for rare variants in sequencing studies (*P*_RARE_ = 0.026). We also identified the gene-wise equivalent of GWS support for *WDR88* (WD repeat-containing protein 88), a gene of unknown function (*P* = 6.5 × 10^−7^). Rare alleles represented on exome chip arrays contribute to the genetic architecture of schizophrenia, but as is the case for GWAS, very large studies are required to reveal additional susceptibility alleles for the disorder.

## Introduction

Schizophrenia is a severe psychiatric disorder with a lifetime risk of ∼1% (1). High heritability points to a major role for inherited alleles in the aetiology of the disorder (1,2); based on recent genomic studies, it appears that the mode of inheritance essentially adheres to a multifactorial model in which the genetic contribution is highly polygenic and in which very large numbers of risk variants (>1000) are involved (1–4). Genome-wide association studies (GWASs) have recently identified over a hundred risk loci, yet cumulatively, under an additive model, these contribute only ∼3.5% of the total population variance in liability (5). Ultimately, around half to a third of the total genetic risk is thought to be indexed by the sort of common alleles present on GWAS arrays (3,6). How the remainder of the genetic liability to schizophrenia is distributed across the allele frequency spectrum is still a matter of controversy, although undoubtedly, rare variants in the form of CNVs, single-nucleotide variants (SNVs) and small insertion deletions are involved (4). The effect sizes of the rare variants that have so far been robustly implicated in the disorder are large, with odds ratios typically ∼2–60 compared with about ≤1.1 for common alleles (7,8). There is no reason why large effect sizes should be a general property of rare alleles; rather, there is likely to be a bias with current studies being underpowered to detect rare alleles that have low-effect sizes.

It is often challenging to draw biological inferences from common variant associations because this class of risk allele appears to primarily exert its effects on regulating mRNA expression or processing rather than through the protein structure (5,9). Another complexity is that it is often not evident which of several possible candidate genes at an associated locus is responsible for a given association. Taken together, this means it is unclear what changes in biological function are likely to be indexed by the association findings. This does not imply that GWAS studies cannot provide the basis for biological insights, indeed common variants have already pointed to several biological processes that are likely involved, including for example the immune system (5).

Relatively few rare variants have been implicated in schizophrenia to date. Nevertheless, studies of this class of variant have been informative for providing clues about pathophysiology. In particular, rare variant studies have highlighted proteins involved in plasticity at glutamatergic synapses, especially the activity-regulated cytoskeleton-associated protein (ARC) and *N*-methyl-d-aspartate receptor (NMDAR) complexes, fragile X mental retardation protein (FMRP) targets, and voltage-gated calcium ion channels (10–13). Individual rare exonic alleles that are of relatively high penetrance may be more informative than their common counterparts in so far as they may be easier to model in animal and cellular systems. These considerations suggest that irrespective of any uncertainty surrounding their overall contribution to heritability, it is a reasonable hypothesis that the pursuit of rare risk variants is an important focus for schizophrenia research. At present, sequencing offers the only systematic approach to doing so in a comprehensive manner, but it is also expensive. In the interim, cheaper ‘exome chip-based’ solutions have been developed to allow exome-focused sampling of a large number of rare exonic variants at a fraction of the cost of sequencing.

Here, we describe what is to date the largest systematic search for uncommon non-synonymous genetic variation in schizophrenia based on 13 688 individuals (5585 cases and 8103 controls) from the UK genotyped using Illumina HumanExome Arrays which are estimated to capture over 95% of the non-synonymous variants that might be detected in an average European ancestry genome through exome sequencing (although it does not cover *de novo*, private, previously unobserved or extremely rare variants; see the ‘Materials and Methods’ section below). The case data set has already been evaluated with respect to common genetic variation as part of the study from the Schizophrenia Working Group of the Psychiatric Genomics Consortium (PGC) (5). Therefore, here, we consider only the results for relatively uncommon variants [minor allele frequency (MAF) < 1%]. Expecting low power to detect individual rare risk alleles even in this sample, we also undertook gene and pathways based tests. To maximize the signal-to-noise ratio for these multi-locus tests, we based our analyses on alleles with MAF < 0.1%, a class of alleles that captured most of the rare variants signal in a recent exome-sequencing study of schizophrenia (10).

## Results

### Single-locus tests

For variants at MAF < 1%, the standardized genomic inflation factor *λ*_1000_ was 0.8788. A quantile–quantile (QQ) plot is given in Supplementary Material, Figure S1. The test statistics are deflated reflecting the presence the low power of regression tests for very rare alleles. Table 1 gives the results for variants at *P* < 1 × 10^−3^, and the results for all variants are given in Supplementary Material, Table S1. No variant was genome-wide significant (GWS). Rare variants (*n* = 3) surpassing this threshold map to GWS loci from the most recent schizophrenia meta-analysis (5) (GWS threshold is *P* < 5 × 10–8). However, these cannot be considered as strong candidates for being pathogenic mutations as they do not even attain a relaxed corrected *P*-value threshold of 2.11 × 10^−5^ (allowing only for the 2371 markers with MAF < 1% that map to the GWS regions). Since at no point in the QQ plot do the test statistics significantly elevate above the null, our single-locus analyses are potentially consistent with a complete absence of association signal captured by rare variation on the present arrays.

**Table 1.** SNV association test results using logistic regression with 10 covariates, limited to *P* < 1 × 10^−3^ and MAF < 1%

SNV	CHR	Position (b37)	MAF (%)	MAF Con (%)	MAF Case (%)	MAF UK Pop (%)	OR	*P*	GWS	ANNOT	Gene
rs140232654	1	111 739 856	0.47	0.35	0.72	0.45	2.12	1.91 × 10^−5^	N	Missense	DENND2D
rs74440117	7	97 863 152	0.16	0.068	0.29	0.19	4.1	6.01 × 10^−5^	N	Missense	TECPR1
rs74892550	16	3 707 191	0.75	0.61	0.99	0.69	1.66	0.00024	N	Missense	TRAP1, DNASE1
rs147802928	19	35 941 518	0.12	0.049	0.22	0.1	4.38	0.0003	N	Missense	FFAR2
rs146838872	9	12 694 094	0.07	0.019	0.16	0.074	9.56	0.0003	N	Missense	TYRP1
rs144338731	2	239 038 917	0.39	0.52	0.25	0.36	0.46	0.00044	N	Missense	ESPNL
rs36059660	20	36 640 870	0.92	0.75	1.2	0.88	1.56	0.00048	N	Missense	TTI1
rs118056333	8	70 515 476	0.78	0.64	1	0.68	1.61	0.0005	N	Missense	SULF1
rs61757580	7	13 737 4693	0.68	0.52	0.89	0.69	1.67	0.00059	Y	Missense	DGKI
rs116932219*	13	49 281 261	0.82	0.98	0.6	0.78	0.61	0.00066	N	Missense	CYSLTR2
rs34703321	12	122 361 711	0.94	1.1	0.73	0.99	0.63	0.00066	N	Missense	WDR66
rs79859029	16	31 927 353	0.24	0.17	0.38	0.2	2.3	0.00077	N	Missense	ZNF267
rs139049409	7	87 005 024	0.16	0.25	0.063	0.13	0.25	0.00078	N	Stop	CROT
rs149406506	10	104 231 098	0.12	0.2	0.036	0.13	0.17	0.0008	N	Missense	TMEM180
rs143085034*	13	49 281 101	0.81	0.97	0.6	0.77	0.61	0.00085	N	Missense	CYSLTR2
rs113141749	17	48 594 980	0.36	0.26	0.5	0.34	1.98	0.00089	N	Missense	MYCBPAP
rs55859133	16	30 128 265	0.16	0.099	0.26	0.17	2.82	0.00095	Y	Missense	MAPK3
rs35890409	12	57 569 339	0.76	0.61	0.95	0.68	1.59	0.00096	Y	Missense	LRP1

GWS indicates whether SNV is within one of the genome-wide significant schizophrenia-associated regions reported by the PGC. Cluster plots for SNVs in this table were visually inspected to ensure correct genotype calling. Positions given are for human genome build 37. Variants marked * are close together on the genome. The minor alleles generally occur in the same samples, indicating that these markers are in strong LD. UK population MAF in column ‘MAF UK Pop’ taken from the UK Exome Chip consortium website (http://diagram-consortium.org/uk-exome-chip/).

For all results at *P* < 0.001, the MAF in the UK population lies between the case and control MAF values, consistent with inflated estimates of effect size at this end of the significance distribution as a result of the winners' curse effect.

### Gene set analysis

Seeking general evidence for an association signal in the exome chip data, we applied a top down approach in which we first examined two large gene-sets (the ‘Materials and Methods’ section) that have been demonstrated to be enriched for schizophrenia susceptibility genes (10,11). We reasoned that if the exome arrays capture any schizophrenia associations, power to detect this should be greater in this more limited, but rationally defined, gene set than in the genome as a whole.

Significance for gene-set analyses of the subsets was corrected by a Bonferroni correction for the number of tests. Both GWAS and RARE subsets were enriched for rare-variant association signal (Table 2). The GWAS set was further assessed for significance using 1000 phenotype-permuted optimized sequence kernel-association test (SKAT-O) analyses, and remained significant (perm *P* = 0.01). This suggests that the exome-focused array does indeed capture schizophrenia associations. Among the six subsets of the RARE set, only the FMRP target set was significantly enriched for rare variant associations, providing further evidence that this set of genes is enriched for rare genetic variation of relevance to schizophrenia. Removing SNVs present in the FMRP target set from the larger RARE set abolished significance of enrichment (*P* = 0.245), suggesting that the signal in the RARE set is driven primarily by the FMRP subset.

**Table 2.** Association analysis of gene sets using SKAT-O, limited to SNVs with MAF < 0.1%

Gene set	*P*	Number of tests	Corr. *P*	Number of genes	Number of SNVs	References
GWAS	0.01	2	0.02	335	1447	(5)
RARE	0.021	2	0.042	1865	17 456	(10–13)
RARE Subsets						
ARC/NMDAR	0.29	6	1	51	294	(12)
ASD/ID de novo	0.26	6	1	665	7306	(11)
Calcium channels	0.38	6	1	19	176	(10)
FMRP targets	0.0048	6	0.029	644	6073	(13)
PSD (human core)	0.0792	6	0.42	401	2774	(10)
SCZ de novo hit genes	0.39	6	1	487	5645	(11)
SECONDARY ANALYSIS						
RARE minus FMRP	0.25	1	0.25	1221	11 385	(10–13)

‘Number of genes' column only includes those genes that contain at least one SNV that passes QC and has MAF < 0.1%. Corr. *P* is the *P*-value, Bonferroni corrected for the number of tests at that level. GWAS set contains genes within the genome-wide significant regions found by the PGC2 schizophrenia study. RARE set contains all genes contained in six subsets—genes relating to the ARC/NMDAR complex, calcium channel genes, FMRP target genes, PSD genes, plus genes hit by *de novo* mutations in schizophrenia or autism and intellectual disability. RARE minus FMRP set contains genes within these subsets other than FMRP target genes.

We tested all individual SNVs and genes within the above gene sets for association with schizophrenia (QQ plot in Supplementary Material, Fig. S2). None was significant after correcting for multiple testing (according to the number of SNVs or genes within each set). The broad gene-set enrichments are, therefore, not being driven by a small number of highly significantly associated SNVs or genes.

We also carried out an exploratory SKAT-O analysis on a combined pathway set consisting of 8680 gene sets taken from the Gene Ontology, Kyoto Encyclopedia of Genes and Genomes, National Cancer Institute, Mouse Genome Informatics, BioCarta, Protein Analysis Through Evolutionary Relationships and Reactome repositories (Supplementary Material, Table S2) (14–24). No gene set approached significance after multiple testing correction.

### Genome-wide association tests

The gene-wide results for all genes with *P* < 0.001 are given in Table 3 and a QQ plot depicting the results of gene-tests of the 12540 genes containing ≥2 non-synonymous SNVs at MAF < 0. 1% is shown in Supplementary Material, Figure S3. A Manhattan plot of the gene results is shown in Supplementary Material, Figure S4, and the complete set of gene results is given in Supplementary Material, Table S3. None of the genes in Table 3 overlap with any of the GWS regions found by the PGC meta-analysis (5). A single gene, WD Repeat Domain 88 (WDR88), reached the gene-wide equivalent of genome-wide significance (*P* < 2.5 × 10^−6^ = 0.05/20 000 correcting for ∼20 000 genes). Details of the seven variants this result is based on are contained in Supplementary Material, Table S4. Note that, two of the variants in the table (exm1453363 and exm1453389) have no minor alleles in one phenotype, and so are assigned *P*-values close to 1 by the single-variant logistic regression association test. SKAT-O is not affected by this issue. Manual inspection of cluster plots confirmed all calls were satisfactory, and multiple rare alleles were not carried by any single individual implying, respectively, that the results are not driven by either by major genotyping error or by non-independence of allele counts due to linkage disequilibrium.

**Table 3.** Gene SKAT-O results (*P* < 0.001) for SNVs with MAF < 0.1%

Gene	Chr	Start (b37)	End (b37)	*P*	Number of SNVs
WDR88	19	33 622 998	33 666 705	6.54 × 10^−7^	7
DSEL	18	65 173 819	65 184 217	2.61 × 10^−5^	13
DNAJC21	5	34 929 698	34 959 069	0.000139	8
DNAH11	7	21 582 833	21 941 457	0.000147	56
THAP4	2	242 523 820	242 576 864	0.000165	5
PIGG	4	492 989	533 985	0.00018	15
MYCL	1	40 361 098	40 367 925	0.000292	2
FAM186B	12	49 981 290	49 999 422	0.000392	17
GAB4	22	17 442 826	17 489 112	0.000661	11
ANGEL2	1	213 165 524	213 189 168	0.000717	2
GPR137	11	64 051 811	64 056 963	0.000734	2

Positions given are for human genome build 37. Cluster plots for SNVs in WDR88 were visually inspected to ensure correct genotype calling.

## Discussion

Most association studies of schizophrenia have focused on common variation, but studies of CNVs and, more recently, studies based on exome sequencing, point to a contribution from rare variants. While rare variation may make only a small contribution to the disorder in comparison with common variants (10), the identification of associated rare non-synonymous variants offers advantages for translating genetic findings into biological understanding by, for example, implicating susceptibility genes more precisely than common variant analysis, or by providing causal mutations that may be more readily modelled in cellular and animal systems.

An important downside of rare variant analysis is cost. It is now clear that large samples on a scale similar to that used in GWAS are, in general, necessary for robustly detecting rare variation (25). However, sequencing, which is required to catalogue most very rare variation, is at least 10 times more expensive than fairly comprehensive GWAS arrays. This has motivated interim measures seeking to identify a proportion of known rare variation using cost-effective genotyping chip alternatives; although they cannot capture private or previously unseen variants, they can capture a proportion of recurrent previously documented variation.

Whether current arrays have potential to capture variation within the frequency spectrum relevant to schizophrenia is unclear given alleles with fairly large effects are under strong selection, and therefore are expected to be of very low frequency (26). A recent sequencing study suggests that most rare variant associations that could potentially be detectable by exome sequencing are found primarily at the very low frequency (MAF < 0.1%) end of the spectrum, a frequency that is unfavourable for current arrays (10). Moreover, supporting that, a study about half the size of the present study and using an array with almost identical content was unable to document any contribution to heritability from rare exonic alleles (27). In this context, our observation of enrichment for the rare-variant association signal in both of the tested primary candidate gene sets is both novel and of practical importance. Those observations suggest that while the total contribution may be very small, rare variants (MAF < 0.1%) present on the exome chip do contribute to the disorder. Hence, our study provides ground for optimism that rare variants in genes can be identified through the application of current exome array products to samples equivalent in size to those in recent GWAS studies. Such studies are already underway under the aegis of the PGC. The findings further suggest that arrays with enhanced exome variant coverage based on larger numbers of exome sequences, particularly from individuals with neuropsychiatric disorders, are likely to be informative.

Within the broad candidate gene categories, the only subcategory that was significantly enriched for the rare-variation association signal (nominally or corrected) was the FMRP target set. The present findings add to what is now strong evidence, based upon multiple study designs, that this gene set is enriched for schizophrenia common variant associations (5) as well as for rare mutations in schizophrenia, autism spectrum disorder (ASD) and intellectual disability (10,11,28–30). Loss of FMRP in Fragile X syndrome results in widespread deficits in synaptic plasticity. Accordingly, our findings extend the burgeoning body of evidence that mechanisms underpinning plasticity are involved in the pathogenesis of schizophrenia.

The other main finding of note in the present study was of the association of a single gene, *WDR88*, at GWS level (corrected for all genes). The function of WDR88 had not been characterized. It belongs to a superfamily of 263 genes of diverse function, only connected by the presence of WDR domains which form β-propeller units at the protein level, giving no clue to the function of WDR88.

The implications of this finding for schizophrenia are therefore unclear. Moreover, as with common variant associations that emerge as GWS in a single study, until it is supported by replication data this finding must be considered provisional.

In summary, the present study provides support for the hypothesis that a proportion of the genetic architecture of schizophrenia is accessible through current genotyping products, supports the broad involvement of targets of FMRP in the disorder, and provides provisional evidence for WDR88 as a candidate schizophrenia susceptibility gene. However, as has been the case with GWAS, very large studies are required to reveal additional susceptibility alleles for the disorder.

## Materials and Methods

### Samples

#### Cases

Our cases were taken from two collections, Cardiff COGS and CLOZUK (31), both of which have been described in the GWAS study from the PGC (5) and which in that study were shown to be typical of schizophrenia with respect to the heritability conferred by common alleles. CLOZUK cases were taking the antipsychotic clozapine. In the UK, patients taking clozapine provide blood samples to allow the detection of adverse drug-effects. Through collaboration with Novartis (the manufacturer of a proprietary form of clozapine, Clozaril), following ethical approval and consistent with the UK Human Tissue Act, we acquired anonymous blood samples from people with treatment-resistant schizophrenia as recorded by the treating psychiatrists on the clozapine registration forms (31). Cardiff COGS cases were recruited from community mental health teams in Wales and England on the basis of a clinical diagnosis of schizophrenia or schizoaffective disorder (depressed sub-type) as described previously (32). After informed written consent, diagnosis was subsequently established using the semi-structured schedules for clinical assessment in neuropsychiatry (33) interview and review of case notes followed by consensus diagnosis according to diagnostic and statistical manual of mental disorders-IV (34) criteria.

#### Controls

Two groups of UK controls were used in this study; UK Blood Service donors (4455 samples) and the 1958 British Birth Cohorts (4615 samples) (35–37). These samples have not been screened for psychiatric illness, which for a disorder with the prevalence of schizophrenia, has negligible impact on power. The study had UK Multicentre Research Ethics Committee approval.

### Genotyping

Genotyping was performed using the Illumina HumanExome and HumanOmniExpressExome BeadChip arrays. Our analyses were restricted to variants present on the HumanExome BeadChip, which in turn is a subset of those on the HumanOmniExpressExome BeadChip. The Illumina Exome BeadChip contains 247 870 genotyping probes, preferentially targeting rare coding variants. The design of the probes was based on over 12 000 sequenced genomes and exomes. Non-synonymous variants that have been observed at least three times (splice and start-stop mutations at least two times) and are observed in two or more data sets were considered for inclusion on the chip with additional custom content (see http://genome.sph.umich.edu/wiki/Exome_Chip_Design for full details).

All cases were typed at the Broad Institute (Cambridge, MA, USA) while the controls were genotyped by the Wellcome Trust Sanger Institute and The Broad Institute. Details of genotyping site and array are given in Supplementary Material, Table S5. The initial sample size was 6991 cases and 9070 controls.

### Analysis

#### Quality control

First pass quality control (QC) was first performed using PLINK on genotypes called using GenCall (38,39). Initial QC for probe base exclusions included Hardy–Weinberg equilibrium (HWE) *P* < 10^−8^, call rate <98%, and non-autosomal location (Supplementary Material, Table S6). Initial QC for subject exclusions were based on call rate <98%, as well as relatedness based on identity by descent analysis, heterozygosity and principal component analysis (PCA) (Supplementary Material, Table S7). PCA was run using Eigenstrat based on 3022 SNPs with MAF > 1% (40,41). Cases and controls were merged with 1100 samples from 11 populations using the HapMap 3 data set (6), and outliers that did not cluster near to the HapMap European individuals were removed to minimize ancestral heterogeneity. In total we excluded 1204 cases, 735 controls and 16 841 markers prior to the zCall post-processing procedure.

zCall is a post-processing step designed to improve the calling of SNVs (42). We applied zCall to each batch (Supplementary Material, Table S5) using batch-specific intensity data. Markers were subsequently excluded if they were monomorphic, had call rates <99%, had HWE *P* < 10^−6^ in any batch, or had a difference in call rate between batches >1%. We also excluded probes where the allele frequencies differed between the two groups of controls (blood donors versus 1958 Birth cohort) at *P* < 0.001, or between the cases assayed on the two types of chip at *P* < 0.0005. These *P*-value thresholds were derived from QQ-plots of the within control and within case analyses. Finally, we excluded variants which did not show a sufficient difference in mean intensity between different genotype clusters (GenTrain score <0.4, cluster separation metric <0.08) (Supplementary Material, Table S8).

We applied a further round of QC to the individuals based on the *Z*-call genotypes, excluding samples on the basis of call rate (>99% required for inclusion), heterozygosity (separately for variants above and <1% MAF) and concordance between database and genetically determined sex (Supplementary Material, Table S9). The final data set comprised 141 204 variants in 5585 cases and 8103 controls.

### Allelic association test

Allelic association testing was performed in PLINK using logistic regression (38). We adjusted for the population structure using the first 10 principle components as covariates. For comparison (but not included in formal analyses), the minor allele frequency for each variant in a sample of 55 276 Europeans from the UK was downloaded from the UK Exome Chip Consortium (http://diagram-consortium.org/uk-exome-chip/).

### Gene-set and gene-wise tests

Variants were allocated to genes according the RefSeq database. Genes and gene sets were examined using the unified method implemented in SKAT-O, where rare effects were tested by finding the optimal linear combination of the burden and SKAT tests (43). As in single-locus analyses, we used ten principal components as covariates to account for population structure within the sample. Analyses were restricted to non-synonymous variants (missense, splice and nonsense) with MAF < 0.1%.

Our primary gene-set hypotheses concerned two broad sets (Table 2) that are enriched for schizophrenia susceptibility genes based on prior independent evidence from either common (GWAS set) or rare (RARE set) variation. The GWAS set contained all genes (*n* = 335) within the 105 autosomal GWS loci reported by the psychiatric genetics consortium (5), excluding the extended multiple histocompatibility complex region which contains hundreds of genes. The RARE set is a large composite candidate gene-set (*n* = 1865) comprising six gene-sets that have been shown in sequencing studies to be enriched for rare mutations in schizophrenia (10,11). These include four gene-sets based upon the biological function: voltage calcium channel genes, genes repressed by FMRP, members of the ARC and NMDAR complexes and genes from the PSD-95 complex. The RARE set also contains a gene-set containing genes affected by *de novo* mutations in schizophrenia case samples fully independent of those used in the current study, and a second gene-set containing genes affected by *de novo* mutations in people with other neurodevelopmental disorders, specifically, ASD and intellectual disability (ID) (10,11,28–30). These disorders were chosen because there is some overlap between associated rare variants in ASD, ID and schizophrenia (11). See Table 2 for summary details and Supplementary Material, Table S10 for gene and SNV membership of the candidate gene-sets.

Tests of individual genes were limited to those with two or more non-synonymous variants with MAF < 0.1%, a total of 12540 genes spanned by 77 446 variants. To obtain an empirical *P*-value for the enrichment for rare variants in the GWAS gene set, we randomly permuted (*n* = 1000) the case/control status of individuals in the Exome chip data set and ran the gene-wide SKAT-O analyses for the GWAS data set for each permutation. It was computationally prohibitive to obtain empirical *P*-values for the larger significant data sets (RARE and FMRP).

### Power

With the proviso of representation on the arrays, and specifying an effect size that is approximately typical of known pathogenic CNVs [odds ratio (OR) = 10] our single-locus tests have power of 90% to detect association to an allele with MAF > 0.04%, and of 25% for MAF > 0.02%. Assuming an OR of 2 (the lowest OR of a known pathogenic CNV), appreciable power (25%) is only obtained at MAF > 0.5% (which is the approximate MAF of the CNV with the OR of 2). Considering SNVs with a MAF of 0.1% and OR 1.6 (the average OR of SNVs with MAF > 0.075% and <0.125% in this study), 80% power is achieved with a sample size of 27 786 cases and 27 786 controls (44). However, these power calculations pertain for each individual locus. Under the hypotheses there are many such loci, power to obtain at least one association of many will be considerably higher.

## Supplementary Material

Supplementary Material is available at *HMG* online.

## Funding

The work at Cardiff University was funded by Medical Research Council (MRC) Centre (G0800509) and Program Grants (G0801418), the European Community's Seventh Framework Programme [HEALTH-F2-2010-241909 (Project EU-GEI)]. Genotyping at the Broad Institute was funded by a philanthropic gift to the Stanley Center for Psychiatric Research. Funding to pay the Open Access publication charges for this article was provided by funds held within Cardiff University for RCUK funded projects.

## Supplementary Material

Supplementary Data
