# Supplementary material for: Exome arrays capture polygenic rare variant contributions to schizophrenia
Source: Hum Mol Genet. 2016 Jan 5;25(5):1001–7. doi: 10.1093/hmg/ddv620 (PMC4754044; doi:10.1093/hmg/ddv620)
Supplement: Supplementary Data [file supp_25_5_1001__index.html]

Exome arrays capture polygenic rare variant contributions to schizophrenia — Exome arrays capture polygenic rare variant contributions to schizophrenia — Supplementary Data 

# Exome arrays capture polygenic rare variant contributions to schizophrenia

## Supplementary Data

Supplementary Data

- Supplementary Data - Doc file
- Supplementary Table 1 - xlsx file
- Supplementary Table 2 - xlsx file
- Supplementary Table 3 - xlsx file
- Supplementary Table 4 - xlsx file
- Supplementary Table 5 - xlsx file
- Supplementary Table 6 - xlsx file
- Supplementary Table 7 - xlsx file
- Supplementary Table 8 - xlsx file
- Supplementary Table 9 - xlsx file
- Supplementary Table 10 - xlsx file
