# Supplementary material for: Exome arrays capture polygenic rare variant contributions to schizophrenia
Source: Hum Mol Genet. 2016 Jan 5;25(5):1001–7. doi: 10.1093/hmg/ddv620 (PMC4754044; doi:10.1093/hmg/ddv620)
Supplement: Supplementary Data [file supp_ddv620_ddv620supp.doc]

Supplemental Figures


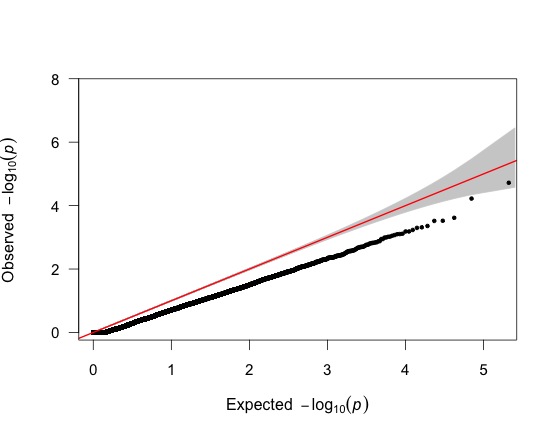


Supplemental Figure 1. QQ plot of post quality control association results with MAF<1%.


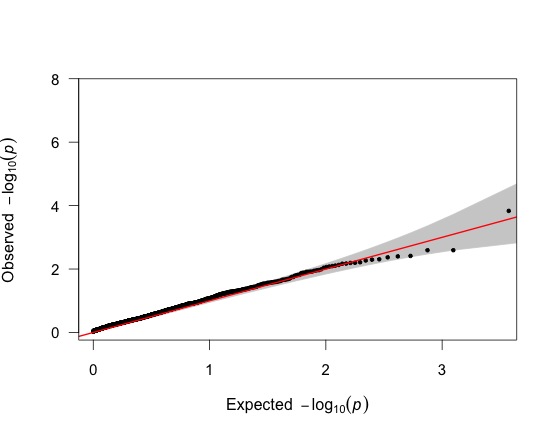


Supplemental Figure 2. QQ plot of SKAT-O gene association results, restricted to genes (N=1847) within the candidate gene sets containing at least two variants at MAF<0.1%.


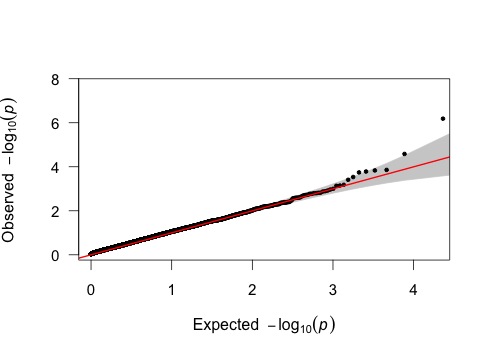


Supplemental Figure 3. QQ plot of SKAT-O all (N=12532) gene association results for genes containing at least two variants at MAF<0.1%.


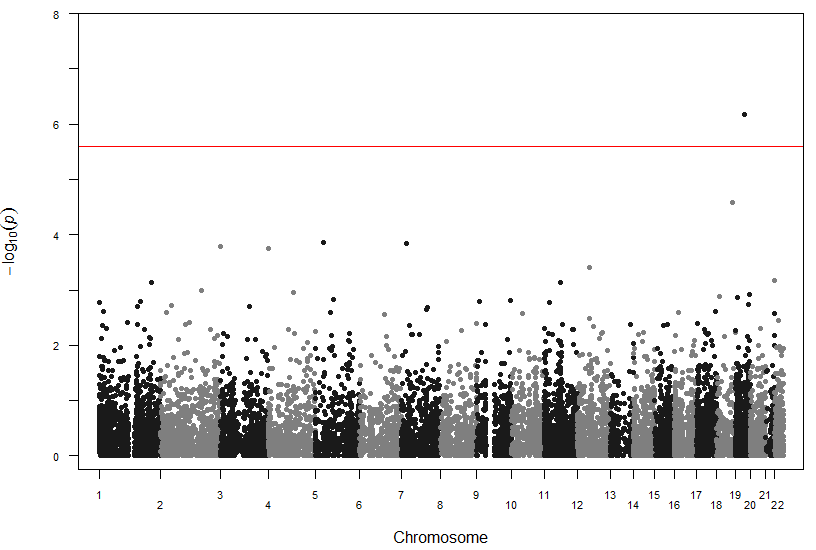


Supplemental Figure 4. Manhattan plot of SKAT-O gene results based on SNVs with MAF < 0.1%

Supplemental Tables

Supplemental Table 1. Single variant association results, MAF < 1%.

Supplemental Table 2. SKAT-O analysis of functional gene sets taken from GO, KEGG, NCI, MGI, BioCarta, PANTHER and Reactome repositories (SKAT-O p<0.01). Gene sets included contained at least 2 variants with MAF<0.1%.

Supplemental Table 3. Complete SKAT-O gene analysis. Genes included contained at least 2 variants with MAF<0.1%.

Supplemental Table 4. Polyphen, SIFT and mutationTaster functional effect predictions for SNVs within WDR88.

Supplemental Table 5. Genotyping platform, initial sample size and initial SNP number by data batch.

Supplemental Table 6. Variant quality control filters for GenCall genotype calls.

Supplemental Table 7. Sample quality control filters for GenCall genotype calls.

Supplemental Table 8. Variant quality control filters for zCall genotype calls.

Supplemental Table 9. Sample quality control filters for zCall genotype calls.

Supplemental Table 10. Gene and variant membership for candidate gene-sets.
